# Bovine NK-lysin peptides exert potent antimicrobial activity against multidrug-resistant *Salmonella* outbreak isolates

**DOI:** 10.1038/s41598-021-98860-6

**Published:** 2021-09-29

**Authors:** Rohana P. Dassanayake, Briony M. Atkinson, Adam S. Mullis, Shollie M. Falkenberg, Eric M. Nicholson, Eduardo Casas, Balaji Narasimhan, Shawn M. D. Bearson

**Affiliations:** 1 Agricultural Research Service, National Animal Disease Center, Ruminant Diseases and Immunology Research Unit, USDA, Ames, IA USA; 2 Agricultural Research Service, National Animal Disease Center, Food Safety and Enteric Pathogens Research Unit, USDA, Ames, IA USA; 3 Department of Chemical and Biological Engineering and Nanovaccine Institute, Iowa State University, Ames, IA USA; 4 Virus and Prion Research Unit, National Animal Disease Center, Agricultural Research Service, United States Department of Agriculture, Ames, IA USA

**Keywords:** Biotechnology, Microbiology

## Abstract

Multidrug-resistant (MDR) *Salmonella* is a threat to public health. Non-antibiotic therapies could serve as important countermeasures to control MDR *Salmonella* outbreaks. In this study, antimicrobial activity of cationic α-helical bovine NK-lysin-derived antimicrobial peptides was evaluated against MDR *Salmonella* outbreak isolates. NK2A and NK2B strongly inhibited MDR *Salmonella* growth while NK1 and NK2C showed minimum-to-no growth inhibition. Scrambled-NK2A, which is devoid of α-helicity but has the same net positive charge as NK2A, also failed to inhibit bacterial growth. Incubation of negatively charged MDR *Salmonella* with NK2A showed increased Zeta potential, indicating bacterial-peptide electrostatic attraction. Confocal and transmission electron microscopy studies revealed NK2A-mediated damage to MDR *Salmonella* membranes. LPS inhibited NK2A-mediated growth suppression in a dose-dependent response, suggesting irreversible NK2A-LPS binding. LPS-NK2A binding and bacterial membrane disruption was also confirmed via electron microscopy using gold nanoparticle-NK2A conjugates. Finally, NK2A-loaded polyanhydride nanoparticles showed sustained peptide delivery and anti-bacterial activity. Together, these findings indicate that NK2A α-helicity and positive charge are prerequisites for antimicrobial activity and that MDR *Salmonella* killing is mediated by direct interaction of NK2A with LPS and the inner membrane, leading to bacterial membrane permeabilization. With further optimization using nano-carriers, NK2A has the potential to become a potent anti-MDR *Salmonella* agent.

## Introduction

*Salmonella* causes an estimated 1.3 million food-borne related illnesses annually^1^, and the Centers for Disease Control and Prevention (CDC) has classified drug resistant *Salmonella* as a serious threat^2^. Multidrug-resistant (resistant to ≥ 3 antimicrobial classes, MDR) *Salmonella* infections have been associated with the consumption of food animal products such as ground beef, chicken, shell eggs, turkey and pork^3^. In 2011, an MDR *S.* Heidelberg outbreak associated with ground turkey caused 136 human illnesses, one death, and the recall of 36 million pounds of ground turkey^4^. In 2015, a multistate outbreak of MDR serovar I 4,[5],12:i- with resistance to ampicillin, streptomycin, sulfisoxazole, and tetracycline (R-type, ASSuT) was linked to the consumption of contaminated pork products, resulting in 188 human infections, 30 hospitalization, and the recall of 523,380 pounds of meat^5^. Currently, thousands of MDR serovar I 4,[5],12:i- sequences have been submitted to the NCBI Pathogens Detection database, with some isolates containing resistance genes for up to 13 antimicrobial classes including antibiotics of clinical importance for treatment of *Salmonella* infections (manuscript in preparation). Global health entities have issued a call to action to “improve strategies for reducing AMR” by developing novel non-antibiotic therapeutics^6^ against MDR *Salmonella* to enhance food safety and public health.

Antimicrobial peptides (AMPs), a diverse group of evolutionarily conserved molecules which are typically composed of 5–50 amino acids^7–9^, could be an important component of such non-antibiotic therapeutics. AMPs feature a broad spectrum of antimicrobial activity and relatively low resistance development^10^. They are produced by most living organisms as a component of their innate immune system and are effective against a broad range of pathogens such as bacteria, protozoa, and fungi^7,9,11^. Based on the amino acid composition and structure, AMPs can be divided into five subgroups: anionic peptides, linear cationic α-helical peptides, cationic peptides enriched for specific amino acids, anionic and cationic peptides that contain cysteine and form disulfide bonds, and anionic and cationic peptide fragments of larger proteins^7^. In addition to antimicrobial activity, AMPs such as cathelicidin peptide LL-37 and chicken NK-lysin show immunomodulatory properties^12,13^.

Bovine NK-lysins are cationic, amphipathic α-helical AMPs produced by T lymphocytes and natural killer (NK) cells that are similar to human granulysin and porcine NK-lysin and are effective against various microbes^14–18^. Amino acid sequence comparisons indicated that granulysin and NK-lysins are related to the saposin-like protein (SAPLIP) family of lipid-binding proteins in lysosomes^16,19,20^. Endsley et al^17^ identified two NK-lysin expression clones (62 and 89) from cultured cattle PBMCs using degenerative primers, and Chen et al^21^ detected four NK-lysin genes (*NK1*, *NK2A*, *NK2B*, and *NK2C*) in bovine genome database search. Tissue specific NK-lysin gene expression patterns of *NK1* and *NK2A* in cattle were highest in the intestinal Peyer’s patches, while *NK2C* expression was almost exclusively limited to the lungs; *NK2B* showed a more general expression pattern^21^. At this time, it is unclear why cattle have four active NK-lysin genes compared to one gene in humans and pigs^14,18,21,22^.

We and others have previously reported the effectiveness of 30-mer cationic, amphipathic α-helical peptides derived from the functional region of bovine NK-lysins against multiple bacterial pathogens^17,20,23–25^. While NK-lysin peptides were highly effective, a differential sensitivity of pathogenic bacteria to four bovine NK-lysin-derived peptides were observed^20,23–25^. Nevertheless, antimicrobial activity of bovine NK-lysin peptides has not been systematically studied against drug-resistant bacteria and MDR *Salmonella* in particular. Cationic AMPs are predicted to bind to bacterial membranes; however, such binding has not been established for bovine NK-lysin peptides. Additionally, although electrostatic attraction between negatively charged bacteria and cationic AMPs are predicted, bacterial Zeta potential changes following bacterial-AMP interactions has also not been reported for bovine NK-lysin peptides.

Exogenous antimicrobial therapeutics frequently display bioavailability deficiencies, requiring frequent dosing to maintain pharmacological efficacy^26^. Additionally, polypeptide agents like AMPs can be susceptible to acidic and protease-mediated proteolysis in the harsh enteric environment. Biodegradable polymer-based nanocarriers could help overcome these shortcomings, enabling an enhanced non-antibiotic anti-MDR *Salmonella* strategy. Polyanhydride nanoparticles composed of sebacic acid (SA), 1,6-bis(*p*-carboxyphenoxy)hexane (CPH), and 1,8-bis(*p*-carboxyphenoxy)-3,6-dioxaoctane (CPTEG) copolymers encapsulate and protect sensitive payloads, navigate challenging biological barriers, and alter payload biodistribution^26–28^. Copolymer chemistry can be modified to tune particle surface erosion and release kinetics, resulting in provision of long-term release to reduce administration frequency^29–31^. Through these mechanisms, polyanhydride nanoparticles enhance payload potency, resulting in a dose sparing effect observed with multiple antimicrobial payloads^32–34^. By controlling AMP release and improving AMP potency, such nanocarriers could form a complementary arm of a novel, non-antibiotic MDR *Salmonella* control strategy.

In the current study, the antimicrobial activity of multiple bovine NK-lysin peptides against MDR *Salmonella* outbreak isolates from swine (SX238), cattle (SX231), and turkey (SB395) was assessed. NK2A was identified as the most effective peptide against MDR *Salmonella* isolates. Therefore, NK2A binding to both LPS and MDR *Salmonella* was further evaluated. Furthermore, payload release kinetics of NK2A encapsulated polyanhydride nanoparticles and anti-MDR *Salmonella* activity of NK2A encapsulated nanoparticles were examined.

## Results

### Antimicrobial activity of NK-lysin peptides against MDR and non-MDR *Salmonella* isolates

**Figure 1:**
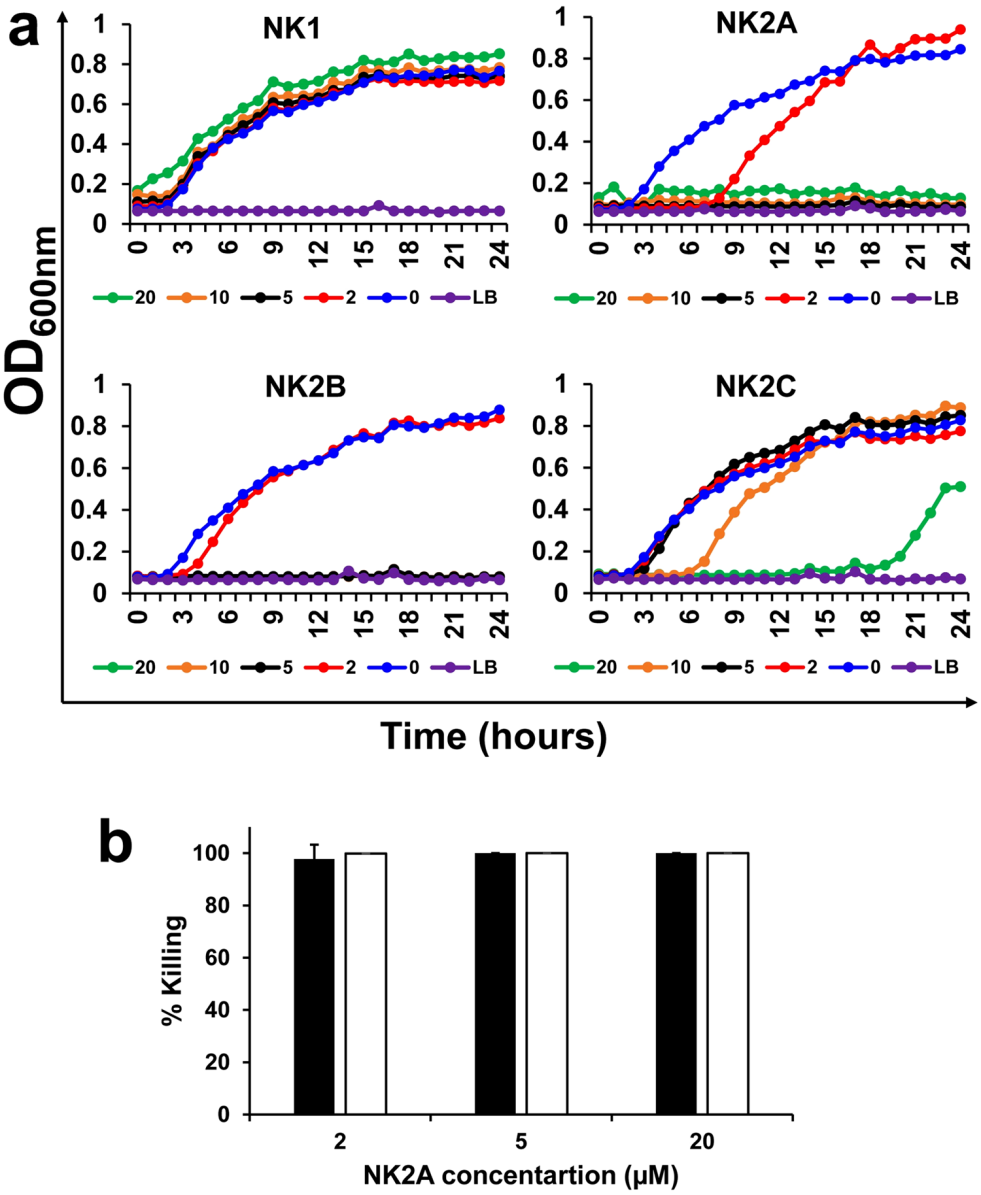
Antimicrobial activity of bovine NK-lysin-derived peptides against MDR *Salmonella*. (**a**) An MDR *Salmonella* outbreak isolate SX238 was incubated with different concentrations of NK-lysin peptides (0, 2, 5, 10 and 20 µM; LB = Luria–Bertani (Lennox broth)), and optical density readings at 600 nm (OD_600nm_) were recorded using an automated growth curve reader. One representative growth graph out of at least three independent experiments is shown. (**b**) Different concentrations of NK2A were incubated with *Salmonella* isolate SX238 at 37 °C for 3 h in PBS (filled bars) or in LB broth (open bars), serially diluted and plated on XLT plates. Following overnight incubation, bacterial colonies were enumerated. Data represent mean percentages of (bacterial) killing ± SD from three independent experiments. *P* = 1.0000 as determined by ANOVA test using GLM procedure from SAS software, Version 9.4 of the SAS System for Windows.

The antimicrobial effectiveness of bovine NK-lysin-derived peptides was assessed against MDR *Salmonella* I 4,[5],12:i:- pork outbreak isolate USDA15WA-1 (SX238) and a non-MDR *Salmonella* isolate (SB377) which is known as universal killer (UK1) (Table 1) in LB broth medium. Four synthetic 30-mer peptides (i.e., NK1, NK2A, NK2B, and NK2C) corresponding to the functional region of bovine NK-lysin (helix-2-loop-helix-3) were tested in growth curve assays. As shown in Fig. 1a and Supplementary Fig. S1, NK1 did not show any *Salmonella* SX238 and SB377 growth inhibition activity at the tested peptide concentrations (highest up to 20 µM, Fig. 1a). Although 10 µM and 20 µM NK2C initially inhibited *Salmonella* SX238 and SB377 growth, bacteria recovered and grew at approximately 7 h and 20 h, respectively (Fig. 1a). In contrast, both NK2A and NK2B were highly effective and completely inhibited growth of *Salmonella* SX238 and SB377 at ≥ 5 µM concentration (Fig. 1a). Since NK2A peptide had a higher antimicrobial activity than NK2B (growth inhibition at 2 µM NK2A concentration for up to 8 h), this bovine NK-lysin peptide was selected for further studies, and the SX238 isolate was chosen for further studies with NK2A. Bactericidal activity of NK2A during 3 h incubation was also tested in PBS and LB broth, and all three NK2A concentrations (2, 5, 20 µM) were highly effective against *Salmonella* SX238*,* with greater than 97% and 99% killing, respectively (*P* = 1.000) (Fig. 1b).

**Table 1:** Sources and properties of MDR and non-MDR *Salmonella* isolates.

*Salmonella* isolates	Source
SX238: MDR *Salmonella enterica* serovar I 4,[5],12:i:- strain USDA15WA-1 Resistant to: ampicillin, streptomycin, sulfisoxazole, and tetracycline	Originally isolated from a 2015 U.S. multi-state pork outbreak from pork products^5^ and sequenced^57^
SX231: MDR *Salmonella enterica* serovar Typhimurium DT193 strain 1434 Resistant to: ampicillin, chloramphenicol, kanamycin, nalidixic acid, streptomycin, and tetracycline	Originally a cattle isolate^58^
SB395: MDR *Salmonella enterica* serovar Heidelberg Resistant to: ampicillin, gentamicin, streptomycin, and tetracycline	Originally isolated from a 2011 ground turkey outbreak^4^ Current isolate was passaged through a turkey^59^
SB377: *Salmonella enterica* serovar Typhimurium isolate UK-1 Resistant to: nalidixic acid (made Nal^R^ in the lab for isolation purposes)	Originally isolated from the spleen of a chick orally inoculated 3 days earlier with a highly virulent *S.* typhimurium isolate retrieved from an infected horse in 1991 Our isolate was passaged through a pig and isolated from the ileocecal lymph node^60,61^

### CD spectroscopy of Sc-NK2A

**Figure 2:**
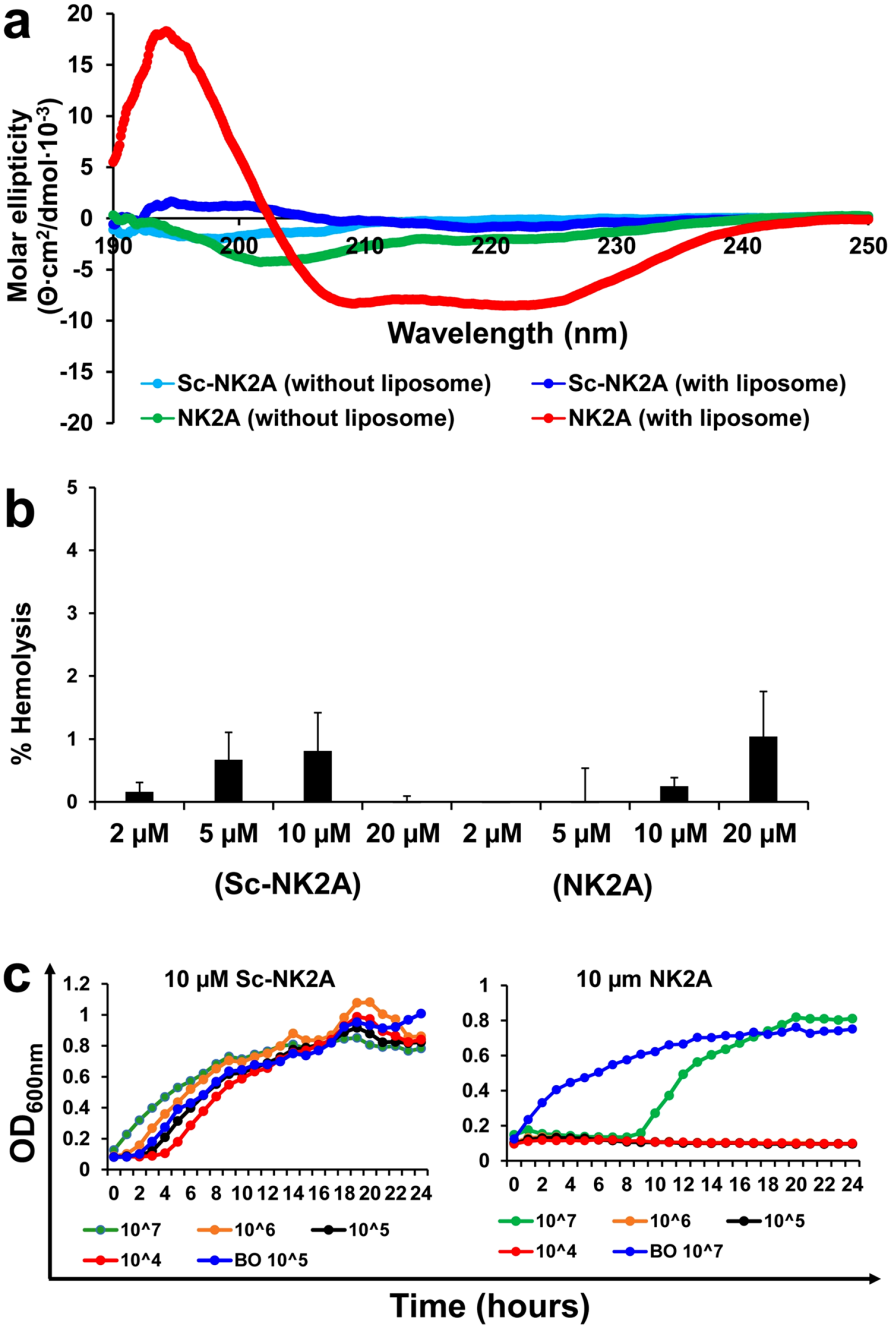
Characterization of scrambled NK2A (Sc-NK2A) peptide. (**a**) Secondary structures of Sc-NK2A and NK2A were determined using circular dichroism (CD) analysis in aqueous buffer (without liposome) and bacterial membrane-mimicking environment (with liposome). Raw data in millidegrees was converted to molar ellipticity (Θ∙cm^2^/dmol∙10^−3^). Average spectra with six accumulations after baseline correction for each of the peptides are shown. (**b**) Hemolytic activities of Sc-NK2A and NK2A on cattle red blood cells (RBCs) were determined spectrophotometrically (OD_405nm_) by measuring released hemoglobin. Data are presented as mean ± SD of percentage of hemolysis from two animals. *P* = 0.1650 as determined by ANOVA test using GLM procedure from SAS software, Version 9.4 of the SAS System for Windows. (**c**). Antimicrobial activity of Sc-NK2A (and NK2A) against *Salmonella* isolate SX238 was recorded using an automated growth curve reader. Optical density readings (OD_600nm_) of different bacterial CFU (10^4^–10^7^ CFU/well; BO = bacteria only) with 10 µM peptide concentrations are shown. Representative graphs out of three independent experiments are shown.

In this study, the secondary structure of scrambled NK2A (Sc-NK2A) in a bacterial membrane-mimicking environment was determined using CD spectroscopy. Previous CD spectral profile analyses confirmed all four bovine NK-lysin peptides to have α-helical conformation when bound with liposomes^24^. The amino acid sequence in NK2A was scrambled and chemically synthesized as a control peptide (Sc-NK2A). Sc-NK2A is predicted to have no α-helical structure but with the same net positive charge as original NK2A (net charge =  + 7.9). Both peptides in phosphate buffer (without liposomes) showed CD spectra indicative of random coil structures (Fig. 2a). NK2A peptide in liposomes showed a CD profile characteristic of predominantly α-helical structure (maximum near ~ 192 nm with double minima near ~ 208 nm and ~ 222 nm) (Fig. 2a). In contrast, Sc-NK2A did not show a CD profile indicative of α-helical structure (Fig. 2a), suggesting that the rearrangement of amino acids resulted in elimination of secondary α-helical structure.

### Cytotoxicity studies with NK-lysin peptides

To assess the toxicity of NK-lysin peptides, a hemolytic assay and a mouse study were performed. It has previously been reported that all four 30-mer NK-lysin-derived peptides have minimal cytotoxic effect against cattle RBCs^24^. To determine whether Sc-NK2A has hemolytic activity, a hemoglobin release assay was performed with cattle RBCs. Both NK2A and Sc-NK2A at the tested concentrations (2–20 µM) showed < 2% hemolytic activity with no significant differences observed between the two peptides (*P* = 0.1650) (Fig. 2b). Mice intraperitoneally injected with 100 µg of NK-lysin peptides (NK1, NK2A, NK2B, NK2C, or Sc-NK2A) did not show signs of toxicity or death during the 2-week observation period. These findings suggest that NK-lysin peptides have minimal toxic effects to both bovine red blood cells and mice at the tested concentrations.

### Antimicrobial activities of Sc-NK2A peptide

**Figure 3:**
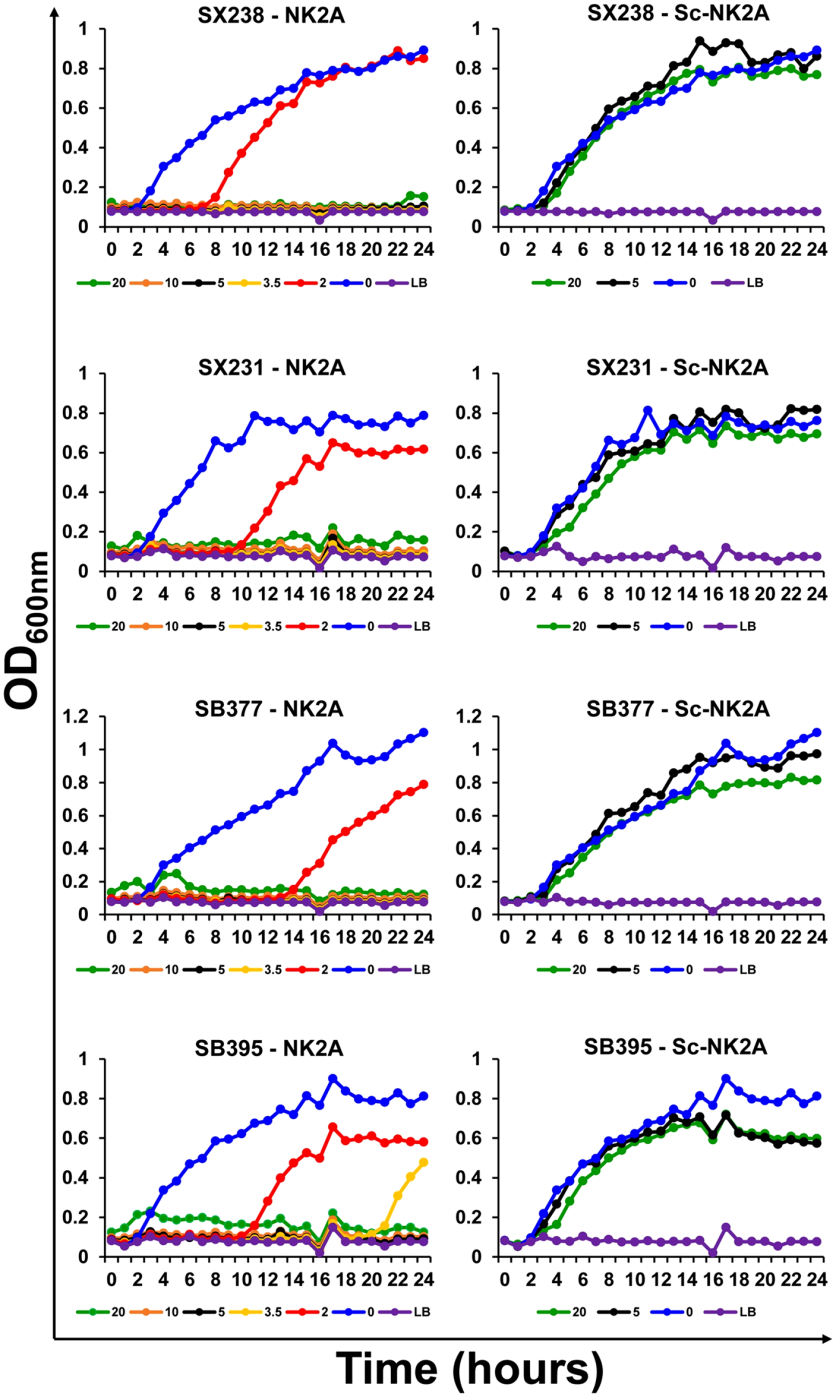
Antimicrobial activity of NK2A and Sc-NK2A against *Salmonella* isolates. Three MDR *Salmonella* outbreak isolates (SX238, SX231 and SB395) and one non-MDR *Salmonella* isolate (SB377) (10^6^ CFU/mL) were incubated with different concentrations of NK2A, Sc-NK2A or Luria–Bertani broth (LB) and optical densities (OD_600nm_) were recorded using an automated growth curve reader over a 24 h period. One representative graph (for each peptide and each isolate) out of at least three independent experiments is shown.

Bacterial growth inhibition assays were performed with *Salmonella* isolate SX238 using both NK2A and Sc-NK2A peptides (Fig. 2c). NK2A completely inhibited growth of SX238 at 10^5–7^ CFU/mL (or 10^4–6^ CFU/well) during a 24 h growth period, and partial growth inhibition was observed (up to 9 h) at 10^8^ CFU/mL (or 10^7^ CFU/well). Sc-NK2A failed to inhibit bacterial growth even at the lowest bacterial concentration (10^4^ CFU/well). Using additional MDR *Salmonella* outbreak isolates originating from cattle (SX231) and turkey (SB395) along with SX238 and SB377 at 10^5^ CFU/well, the antimicrobial activity of NK2A and Sc-NK2A was compared. NK2A inhibited the growth of the four *Salmonella* isolates at or above the 5 µM concentration, although three of the four isolates were sensitive to a final NK2A concentration of 3.5 µM (Fig. 3). However, even 20 µM Sc-NK2A failed to show *Salmonella* growth inhibition activity (Fig. 3).

### Effect of NK2A on Zeta potentials of MDR *Salmonella*

Bacterial Zeta potential has recently been shown to serve as a proxy for membrane permeabilization^35^. Accordingly, Zeta potential measurements of NK-lysin peptides and *Salmonella* were evaluated. Cationic NK-lysin peptides including Sc-NK2A showed positive Zeta potential values in the range of 16.3–24.8 mV (Table 2). In contrast, three MDR and one non-MDR *Salmonella* isolates had negative Zeta potential values, which is attributed to the anionic LPS-outer membrane (Table 3). Bacterial Zeta potential changes following incubation with NK2A (2 µM for 20 min) were assessed. Alteration of *Salmonella* Zeta potentials was observed following incubation with NK2A; the negative Zeta potentials of all four *Salmonella* isolates changed to positive Zeta potential values (ranging from 6.1–16.0 mV) suggesting NK2A-LPS (*Salmonella*) and/or NK2A-cytoplasmic membrane binding (Table 3). MDR *Salmonella* isolate SB395 with 2 µM Sc-NK2A increased Zeta potential value (7.8 mV), indicating that Sc-NK2A also bound to *Salmonella* membranes (Table 3). However, as shown in Fig. 3, Sc-NK2A at 20 µM did not inhibit *Salmonella* growth, suggesting that the scrambled, non-pore forming peptide can associate closely with the cell surface to change the observed bacterial surface charge to positive, but the presence of the α-helical structure is critical for anti-bacterial activity.

**Table 2:** Characterization of bovine NK-lysin peptides and polyanhydride nanoparticles. NA not applicable.

Reagent/formulation	Mean diameter (nm)	Zeta potential (mV)	Encapsulation efficiency
NK2A	NA	+ 24.8 ± 1.1	NA
Sc-NK2A	NA	+ 16.3 ± 6.2	NA
NK1	NA	+ 23.5 ± 2.3	NA
NK2B	NA	+ 17.0 ± 1.7	NA
NK2C	NA	+ 17.8 ± 2.2	NA
Empty 20:80 CPTEG:CPH	239 ± 96	− 21.4 ± 3.5	NA
Empty 10:90 CPTEG:SA	334 ± 127	− 33.5 ± 1.0	NA
Empty 20:80 CPH:SA	341 ± 139	− 29.8 ± 0.9	NA
20:80 CPTEG:CPH 5% NK2A	243 ± 97	+ 20.7 ± 2.9	34.3%
20:80 CPTEG:CPH 5% kanamycin	285 ± 91	+ 39.8 ± 4.4	42.1%
10:90 CPTEG:SA 10% NK2A	367 ± 156	+ 37.2 ± 2.5	47.3%
20:80 CPH:SA 10% NK2A	641 ± 150	+ 39.5 ± 3.0	38.9%

**Table 3:** Zeta potential of MDR and non-MDR *Salmonella* outbreak isolates before and after 2 µM NK2A and Sc-NK2A treatments for 20 min. NT not tested.

	SX238	SX231	SB377	SB395
H_2_O/PBS	− 2.8 ± 0.7 mV	− 6.3 ± 2.1 mV	− 9.0 ± 3.1 mV	− 4.0 ± 1.7 mV
NK2A	+ 16.0 ± 1.5 mV	+ 15.8 ± 1.2 mV	+ 13.3 ± 1.9 mV	+ 6.1 ± 2.7 mV
Sc-NK2A	NT	NT	NT	+ 7.8 ± 1.4 mV

### Effects of NK2A on MDR *Salmonella* morphology

**Figure 4:**
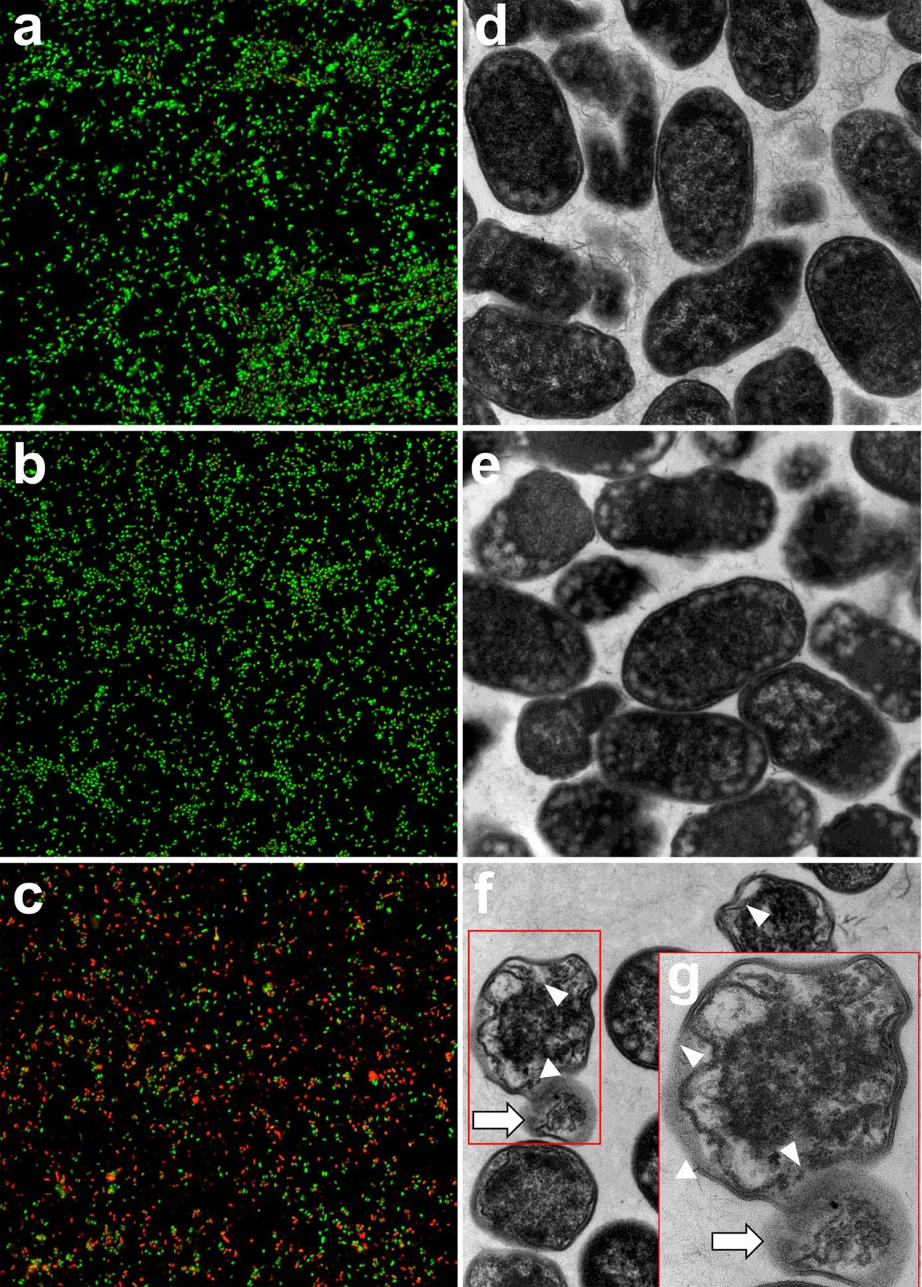
Morphological changes of *Salmonella* following incubation with NK2A and Sc-NK2A. Confocal laser scanning microscopy (CLSM) was performed to identify live (green, SYTO 9) and dead (red, PI) *Salmonella*. CLSM images of *Salmonella* outbreak isolate SX238 incubated with (**a**) PBS, (**b**) 5 µM Sc-NK2A, and (**c**) 5 µM NK2A. Transmission electron microcopy (TEM) was performed to visualize damage to the membranes of MDR *Salmonella* outbreak isolate SX238. TEM images of MDR *Salmonella* SX238 incubated with (**d**) PBS, (**e**) 20 µM Sc-NK2A, and (**f**) 20 µM NK2A (**d**–**f** = 23,000 × magnification). Dead bacteria with damaged outer- and inner-membranes [(**f** and **g**), white triangles], loss of normal bacterial morphology and cytosolic contents (g = 68,000 × magnification, white arrow) were visible only in NK2A treated samples. Representative images out of three independent experiments are shown.

To morphologically evaluate the dead MDR *Salmonella* SX238 following incubation with NK2A, confocal laser scanning microscopy (CLSM) and transmission electron microscopy (TEM) studies were performed. The LIVE/DEAD (*Bac*Light) bacterial viability staining method was used to differentiate live (stained with SYTO 9, green) from dead (stained with propidium iodide (PI), red) bacteria. Primarily SYTO 9-stained bacteria (green) and only a few PI-stained bacteria (red) were observed in both control (PBS; Fig. 4a) and Sc-NK2A (Fig. 4b) treated samples, indicating that Sc-NK2A did not have detectable antimicrobial activity. In contrast, many dead (red) *Salmonella* SX238 were detected in NK2A treated samples, further confirming the growth assay findings that NK2A is bactericidal to *Salmonella* (Fig. 4c).

To visualize ultrastructural changes to *Salmonella* SX238 outer- (LPS) and inner- (cytoplasmic) membranes following incubation with NK2A, bacterial samples were evaluated using TEM. *Salmonella* SX238 in PBS (control, Fig. 4d) or with Sc-NK2A (Fig. 4e) were normal bacilli-shaped bacteria with intact outer- and inner-membranes and typical electron-dense cytoplasm. The periplasmic spaces in both samples were thin and uniform. These observations suggest that the bacteria were alive, and Sc-NK2A did not cause detectable damage to the bacterial membranes. In contrast, SX238 treated with NK2A had extensively damaged outer- and inner-membranes and cytosolic content leakage (Fig. 4f,g). Due to the loss of membrane integrity and cytosolic contents, the bacteria were smaller in size, lacked the typical bacilli morphology and appeared collapsed. NK2A-treated SX238 had slightly wavy inner membranes (Fig. 4f,g), wider periplasmic spaces (Fig. 4f,g and 6d) and were visibly non-uniform. Collectively, these findings suggest that NK2A disrupted both the outer- and inner-membranes of *Salmonella*.

### Kinetics of MDR *Salmonella* killing by NK2A

**Figure 5:**
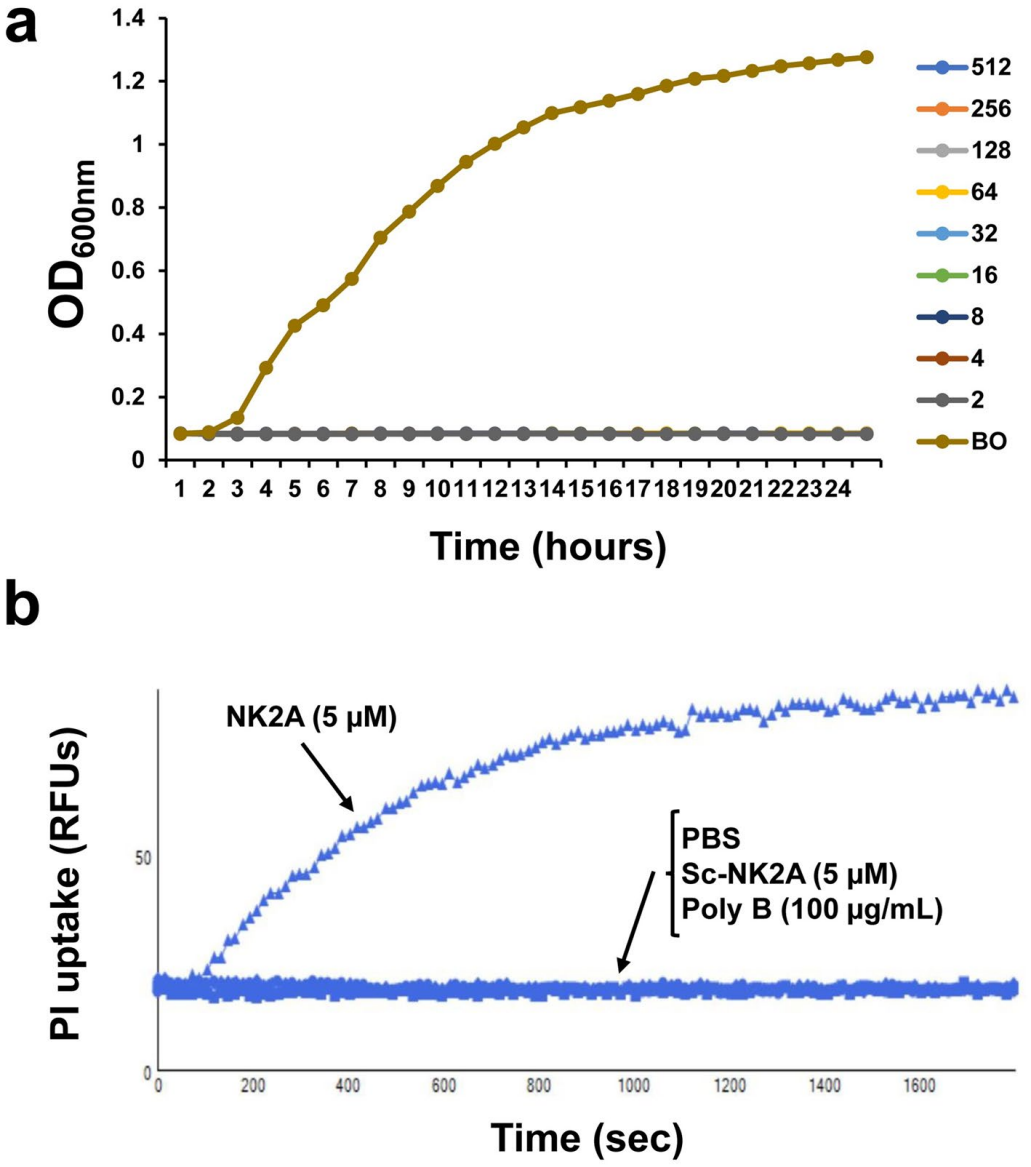
Assessment of propidium iodide (PI) uptake by *Salmonella* incubated with NK2A, Sc-NK2A and polymyxin B. (**a**) Growth curve graphs (OD_600nm_) were generated using an automated growth curve reader for *Salmonella* outbreak isolate SX238 after incubation with different concentrations of polymyxin B (0–512 μg/mL). One representative graph out of three independent experiments is shown. (**b**) *Salmonella* (SX238) was preincubated with PI followed by the addition of NK2A, Sc-NK2A (5 μM final concentration) or polymyxin B (100 μg/mL concentration), and PI signal was measured by a fluorescent microplate reader using flex mode with 15 s intervals for up to 30 min. PI uptake signals are shown as relative fluorescent units (RFUs). Bacteria incubated with PBS was used as a negative control. One representative PI uptake graph out of three independent experiments is shown.

The bacterial killing, CLSM and TEM studies suggest that the bactericidal effect of NK2A against *Salmonella* is membranolytic activity. To determine the kinetics of *Salmonella* cytosolic membrane permeabilization by NK2A, a PI uptake assay was performed. PI is a cell impermeant fluorescent dye and can only bind/intercalate with cellular DNA (and RNA) when the bacterial cytoplasmic membranes are compromised^20^. With similar features as NK2A, cyclic polycationic polymyxin B was selected as a positive control for the assay because polymyxin B is a peptide and a rapid-acting bactericidal antibiotic which interacts with bacterial LPS as well as the cytoplasmic membrane^36^. Growth curve assays with polymyxin B (2–512 µg/mL) showed that as little as 2 µg/mL was sufficient to completely inhibit *Salmonella* SX238 growth during the 24 h incubation (Fig. 5a). No PI uptake signal was observed with *Salmonella* SX238 incubated with polymyxin B at concentrations even as high as 100 µg/mL during a 30 min observation period (Fig. 5b) as well as 6 h observation period (Supplementary Fig. S2). Furthermore, *Salmonella* incubated with Sc-NK2A or PBS (negative control) did not show PI uptake signals (Fig. 5b), indicating that neither polymyxin B nor the scrambled NK2A peptide disrupted the cytosolic membranes. In contrast, rapid PI uptake was observed for *Salmonella* SX238 incubated with NK2A (5 µM), and PI fluorescent signal intensity steadily increased during the 30 min incubation period, suggesting that the bacterial cytoplasmic membrane was disrupted (or permeabilized) by NK2A (Fig. 5b).

### Binding of NK2A with *Salmonella* LPS

**Figure 6:**
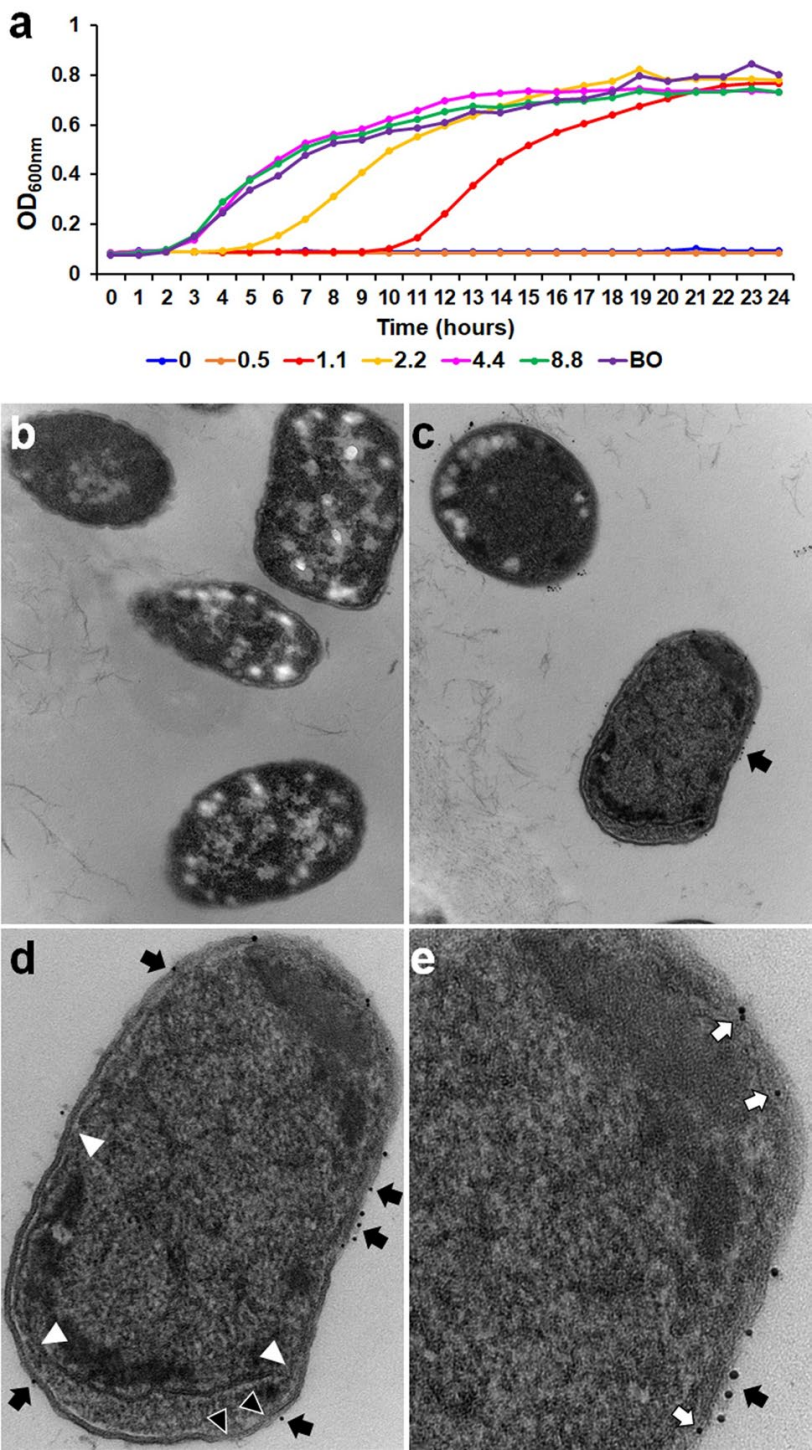
NK2A binding to *Salmonella* lipopolysaccharides (LPS). (**a**) Affinity of NK2A to *Salmonella* LPS was assessed by bacterial growth assay. Five µM (or 2.2 μg) NK2A was preincubated with different concentrations of *Salmonella* LPS (0.5–8.8 μg) for 30 min prior to incubation with MDR *Salmonella* outbreak isolate SX238, and growth was recorded for 24 h using an automated growth curve reader (OD_600nm_; BO = bacteria only). One representative graph out of three independent experiments is shown. (**b**–**e**) TEM images were obtained for *Salmonella* outbreak isolate SX238 incubated with control AuNPs (**b**) or 20 μM NK2A-AuNPs (**c**–**e**). Black arrows indicate NK2A-AuNPs bound to MDR *Salmonella* LPS (outer membrane) and white arrows indicate NK2A-AuNPs bound to the inner cytoplasmic membrane. White triangles indicate damaged inner cytoplasmic membranes and black triangles indicate damaged outer membranes (LPS; **d**). A larger periplasmic space was observed in the bacteria incubated with NK2A-AuNPs compared to control AuNPs. (b/c = 30,000 × magnification; d = 68,000 × magnification; e = 120,000 × magnification). Representative images out of two independent experiments are shown.

To visualize NK2A binding to *Salmonella* LPS and assess concordant NK2A-induced *Salmonella* growth inhibition, a bacterial growth curve assay was performed following preincubation of NK2A (5 µM or 2.2 µg) with increasing LPS concentrations (0, 1.1, 2.2, 4.4 and 8.8 µg). As previously shown (Fig. 1 and 3), 5 µM NK2A was able to completely inhibit the growth of *Salmonella* SX238 during 24 h incubation (Fig. 6a). Preincubation of 5 µM NK2A with 1.1 µg and 2.2 µg LPS showed delayed bacterial growth recovery (~ 5–10 h), while LPS at 4.4 µg and 8.8 µg completely inhibited the antimicrobial activity of NK2A on *Salmonella* SX238 (Fig. 6a). These findings suggest that NK2A irreversibly binds to LPS.

To confirm this binding, *Salmonella* SX238 was incubated with NK2A conjugated with gold nanoparticles (NK2A-AuNP) (10 nm diameter) or control AuNPs (without NK2A) and investigated for particle-LPS localization and membrane integrity by TEM. No AuNP localization with *Salmonella* SX238 membranes were observed in AuNP control samples (Fig. 6b). By contrast, NK2A-AuNPs produced numerous *Salmonella* membrane localization sites (Fig. 6c). Most of the NK2A-AuNPs were bound to *Salmonella* outer membrane or LPS (Fig. 6c–e; black arrows), while fewer NK2A-AuNPs bound to the inner cytoplasmic membranes (Fig. 6e, white arrows). Bacteria with damaged outer LPS (Fig. 6d, black triangles) and inner cytoplasmic membranes were observed (Fig. 6d, white triangles), and these bacterial cells had expanded periplasmic spaces (Fig. 6d), presumably due to NK2A-mediated rupture of the inner cytoplasmic membrane.

### Characterization of nanoparticles

**Figure 7:**
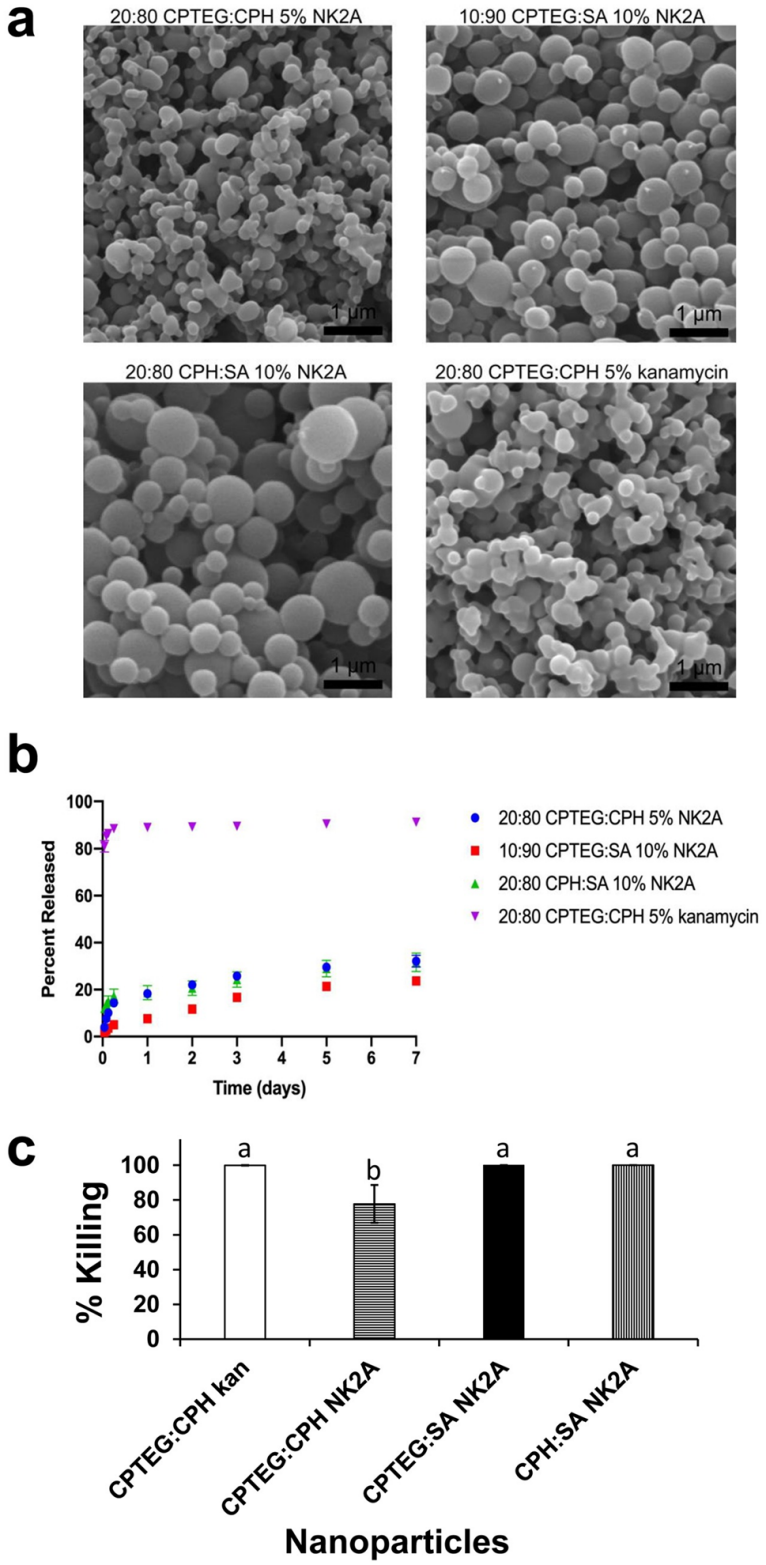
Characterization and killing efficacy of polyanhydride nanoparticles. (**a**) SEM micrographs of NK2A and kanamycin loaded polyanhydride nanoparticles. (**b**) Nanoparticle release kinetics of NK2A and kanamycin in PBS, pH 7.4 were determined by microBCA assay or *o*-pthalaldehyde derivatization followed by spectroscopy, respectively. (**c**) *Salmonella* outbreak isolate SX238 was incubated with NK2A (final concentration =  ~ 5 μM), kanamycin (kan; final concentration =  ~ 16 μg/mL) nanoparticles, or empty nanoparticles (negative control) for 3 h. Samples were serially diluted in LB broth, plated on to XLT plates, and bacterial colonies were enumerated after overnight incubation. Percentages of bacterial killing are presented as mean ± SD from three independent experiments. Bars with no common letters (a and b) differ significantly (*P* < 0.0001) as determined by ANOVA test using GLM procedure from SAS software, Version 9.4 of the SAS System for Windows.

To test the compatibility of NK2A with polymeric nano-carrier delivery devices, a small library of polyanhydride nanoparticle formulations was synthesized. Empty 20:80 CPTEG:CPH, 10:90 CPTEG:SA, and 20:80 CPH:SA nanoparticle formulations had mean diameters of ~ 239–341 nm (Table 2). NK2A-loaded formulations had similar size distributions to the corresponding empty formulations (Table 2). Morphologically, the CPTEG:SA and CPH:SA nanoparticles tended to be spherical and less aggregated, while the CPTEG:CPH nanoparticles tended to be less spherical and slightly more aggregated when visualized by SEM (Fig. 7a).

Payload type dramatically affected nanoparticle release kinetics in PBS, pH 7.4. Kanamycin-loaded nanoparticles showed a burst-dominated release profile, with approximately 90% release within 1 day and little drug released over the following 6 days (Fig. 7b). This result could indicate poor compatibility of the relatively hydrophilic drug within the hydrophobic polyanhydride polymer matrix, resulting in kanamycin localization at the particle surface. By contrast, NK2A formulations showed a slow, sustained release, releasing < 35% of their payload over 1 week (Fig. 7b). This slow release is likely a result of the hydrophobicity of the NK2A peptide enabling better mixing with the polymer within the nanoparticles. This correlates with a more homogeneous distribution of NK2A within the particles, allowing polymer degradation and surface erosion to control release. All NK2A- and kanamycin-loaded nanoparticles showed moderate encapsulation efficiencies between 34.3 and 47.3%.

Each empty nanoparticle chemistry showed negative Zeta potential values, attributable to dicarboxylic acid residues that are produced at the surface after hydrolysis of the anhydride bond^37^ (Table 2). However, NK2A encapsulation produced a profound, sign-inverting increase in observed Zeta potentials, likely due to the cationic nature of the NK2A peptide (Table 2).

#### Antimicrobial activity of NK2A nanoparticles

To assess the antimicrobial activity of NK2A nanoparticles, MDR *Salmonella* SX238 (10^6^ CFU/ml) was incubated (3 h) with NK2A nanoparticles (5 µM final concentration). Efficient *Salmonella* SX238 killing was observed with 20:80 CPTEG:CPH kan nanoparticles (final kanamycin concentration = 16 µg/mL) (Fig. 7c). Both 10:90 CPTEG:SA and 20:80 CPH:SA NK2A-loaded nanoparticles showed 100% *Salmonella* SX238 killing, while 20:80 CPTEG:CPH NK2A-loaded nanoparticles exhibited ~ 80% SX238 killing (Fig. 7c) (*P* < 0.0001). This differential *Salmonella* SX238 killing could indicate polymer chemistry interactions with the NK2A peptide, affecting its capacity to localize at the bacterial surface, or alternatively polymer-bacteria interactions. These considerations should be explored further in future research.

## Discussion

*Salmonella* is a major causative agent of bacterial foodborne illness in the U.S.^3,38^. In a 2019 United States antibiotic resistance threats report, the CDC classified both drug resistant nontyphoidal and typhoidal *Salmonella* as serious threats to global health^2^. Approximately 10% of *Salmonella* are multidrug-resistant with a few of the isolates defined as extremely resistant (resistant to eight or more antimicrobial classes^39^); thus, innovative non-antibiotic strategies are needed to limit *Salmonella* colonization of food animals and contamination of their food products^40^. Despite the availability of over 19,000 antimicrobial agents^41^, the literature on MDR *Salmonella* susceptibility to AMPs is notably sparse^42^. AMPs are ideal molecules to test against MDR *Salmonella* because AMPs generally induce rapid bacterial killing, have a broad spectrum of antimicrobial activity, and have a reduced proclivity for cultivating bacterial resistance compared to classical antibiotics^10^. It has previously been reported that 30-mer peptides derived from bovine NK-lysins are potent inhibitors against a variety of bacteria^17,20,21,23–25^. This study established NK2A and NK2B as highly effective against *Salmonella* isolates (MDR and non-MDR). Both peptides were able to inhibit the growth of *Salmonella* at low micromolar concentrations (3.5–5 µM) during a 24 h incubation in LB broth (Fig. 1a). Although certain AMPs show reduced antimicrobial activity in the presence of salt^12,13,43,44^, NK2A and NK2B were highly effective in both LB broth and PBS containing physiological concentrations of salt (NaCl ~ 100–150 mM).

The antimicrobial activity of cationic AMPs is predicted to be due to nonspecific interaction with bacterial membranes^7^. Bovine NK-lysin peptides are cationic α-helical AMPs and are expected to electrostatically interact with negatively charged bacterial membranes, resulting in pore formation, intracellular content leakage and bacterial death^20,21^. The net positive charges of bovine NK-lysin peptides in physiological buffer (PBS) is predicted to be + 5.0 to + 7.9. In general, bacterial surface charge or Zeta potential is negative due to the presence of negatively charged lipopolysaccharides in Gram negative bacteria and teichoic acids in Gram positive bacteria. Due to the complex nature of Gram negative bacterial LPS molecules, interactions of AMPs and LPS are poorly understood. Phosphatidylethanolamine, phosphatidylglycerol and cardiolipin are the major lipid components of bacterial cytoplasmic membranes among which the latter two are negatively charged^45^. Using a Zetasizer, it was confirmed that NK-lysin peptides have net positive Zeta potentials while *Salmonella* isolates have net negative Zeta potentials (Tables 2–3). Incubation of *Salmonella* isolates with NK2A resulted in increased bacterial Zeta potentials, suggesting electrostatic interaction and charge neutralization between NK2A and bacterial LPS and cytoplasmic membrane. Some studies suggested that increased Zeta potentials can be used as a potential marker to assess damage to bacterial membranes or viability^46^. However, increased Zeta potentials of *Salmonella* (isolate SB395) were observed even with Sc-NK2A which has minimal bacterial killing activity. Therefore, the findings suggest that increased bacterial Zeta potential alone is an imperfect tool for assessing AMP-mediated membrane permeability of *Salmonella* isolates.

Although direct bovine NK-lysin peptide binding to bacterial membranes has not been demonstrated, the increase in Zeta potential of *Salmonella* with NK2A in this study suggested NK2A-membrane interactions. Membranolytic activity of NK2A is supported by our experimental findings from live/dead bacterial viability staining, PI uptake assay, and TEM. Moreover, two specific experiments were conducted to confirm NK2A binding with MDR *Salmonella* SX238 membranes. Preincubation of NK2A with increasing concentrations of *Salmonella* LPS prevented NK2A-mediated MDR *Salmonella* SX238 growth inhibition (Fig. 6a). This suggests that NK2A-LPS binding is irreversible. TEM detection of NK2A-AuNP conjugates on both bacterial outer- and inner-membranes confirmed direct binding of NK2A to MDR *Salmonella* SX238 LPS and the cytoplasmic membrane.

Our ultimate goal is to develop non-antibiotic strategies to control *Salmonella* colonization and shedding in food animals. Of the four bovine NK-lysin peptides tested in this study, NK2A appeared to be the best candidate. In general, AMPs show poor pharmacokinetic properties because of their small size, hydrophobicity, and sensitivity to proteolytic degradation. Polyanhydride nanoparticles have been extensively characterized as a delivery platform for small molecules (including antimicrobials) and biomolecules^29,30,32,47–51^. Polyanhydride nanoparticles entrap and protect sensitive payloads from harsh physiological environments (like proteases and low pH), traffic the payload to the site of action, and control the release of the payload^27,28^. These features could improve peptide stability, enhance AMP bioavailability, reduce proteolytic degradation, and decrease treatment frequency. In vitro release kinetics assays revealed that all tested nanoparticle formulations produced slow and sustained release of NK2A. These nanoparticle formulations also preserved NK2A functional activity, given that ~ 80% (in the case of 20:80 CPTEG:CPH particles ) to 100% (for 10:90 CPTEG:SA and 20:80 CPH:SA particles) MDR *Salmonella* killing was observed. These findings suggest that polyanhydride nanoparticles have the features of a suitable nano-carrier delivery platform for NK2A, and merit further investigation in future animal trials.

In conclusion, this study revealed that among four bovine NK-lysin peptides, NK2A and NK2B showed potent antimicrobial activity against *Salmonella* isolates (MDR and non-MDR). Using a variety of techniques, it was demonstrated that NK2A directly and irreversibly binds to bacterial LPS and the cytoplasmic membrane, leading to membrane permeability, intracellular content leakage, and bacterial death. Initial investigation of NK2A-loaded polyanhydride nanoparticles indicates both sustained release and peptide protection capabilities, motivating further exploration of a nano-carrier delivery approach for NK-lysin-based therapies to treat MDR *Salmonella* infections. Such an innovative, non-antibiotic strategy could aid efforts to control the spread of MDR *Salmonella* infections in animal and human populations.

## Materials and methods

### Peptide synthesis

Thirty-mer peptides corresponding to the functional region helices 2-loop-3 of bovine NK-lysins (NK1: VIIHVTSKVCSKMGLWSILCNQMMKKYLNR, net charge =  + 5.0; NK2A: TVIEVASKMCSKMRLLKGLCKSITKRFLRR, net charge =  + 7.9; NK2B: TVIEAASKVCGKMGPLKGLCKSITKRFLRR, net charge =  + 6.9; NK2C: TVIEEASKVCSKMRLLKGLCKSIMKKFLRT, net charge =  + 5.9 and a scrambled version of NK2A (Sc-NK2A): MFVSLSLATIRLTGKSKCKKERCMILKRRV, net charge =  + 7.9) were synthesized by FMOC solid-phase peptide synthesis chemistry, aliquoted (1 mg/tube), and supplied as trifluoroacetate salt with over 95% purity (Peptide 2.0 Inc., Chantilly, VA). Lyophilized peptides were dissolved in Dulbecco’s phosphate-buffered saline (PBS, pH 7.2), aliquoted, and stored at − 20 °C until used.

### Peptide concentration determination

Concentrations of all NK-lysin peptides and Sc-NK2A were determined by quantitative amino acid analysis at the Protein Chemistry Laboratory, Texas A&M University and the Center for Biotechnology, University of Nebraska-Lincoln.

### Animals and ethics

The authors complied with the ARRIVE guidelines. All animal procedures were conducted in strict accordance with the Animal Welfare Act, and all procedures were approved by the Institutional Animal Care and Use Committee at the National Animal Disease Center, Ames, IA. Blood samples collected from 4 years old healthy two Holstein cows from National Animal Disease Center were used to isolate red blood cells.

### Mouse study

To determine whether NK-lysin peptides show in vivo cytotoxic effects, a mouse study was conducted. Briefly, thirty 5–6 weeks old female Swiss-Webster mice (Envigo, Indianapolis, IN) were housed in groups in six individual cages (5 mice per cage) in ABSL2 facilities with ad libitum access to food and water in a 12-h light:dark cycle. After 1 week of acclimation, mice were intraperitoneally injected with a single dose of 100 µg of NK-lysin peptides (NK1, NK2A, NK2B, NK2C, or Sc-NK2A, ~ 4 mg/kg of body weight) in PBS (1 mL). Mice injected with PBS (1 mL) served as a negative control for the study. Mice were daily observed for any signs of toxicity and were euthanized 2 weeks post peptide administration.

### Circular dichroism (CD) analysis

CD analysis of all four bovine NK-lysin peptides were previously described^24^. Therefore, CD analyses were performed here using the same methods for NK2A and Sc-NK2A in potassium phosphate buffer and liposomes using Jasco J-815 CD spectrophotometer (Jasco, Easton, MA).

### Hemolytic activity assay

The hemolytic activity of all four bovine NK-lysin peptides, as the amount of hemoglobin released by the lysis of cattle red blood cells (RBCs), were previously determined^20^. Therefore, only hemolytic activity of Sc-NK2A was compared with NK2A (final concentrations for both peptides at 2, 5, 10, and 20 µM) using cattle RBCs in this study as described previously^20^. Triton X-100 and PBS were used as positive and negative control for the assay, respectively.

### Nanoparticle synthesis

SA monomer was purchased from Sigma Aldrich (St. Louis, MO). CPH and CPTEG monomers were synthesized as previously described^52,53^. The 20:80 CPTEG:CPH, 20:80 CPH:SA, and 10:90 CPTEG:SA copolymers were synthesized by melt condensation to number average molecular weights of approximately 7.5, 17.5, and 17.5 kDa, respectively^49,52–54^, and composition and molecular weight were confirmed by ^1^H NMR analysis using a Varian MR-400 (Varian, Inc., Palo Alto, CA). Nanoparticles were synthesized by flash nanoprecipitation^29^. Nanoparticles were collected by vacuum filtration, scanning electron microscopy (SEM, FEI Quanta 250, Hillsboro, OR) was used to image all formulations, and size distributions were calculated using Fiji image analysis software^55^. Nanoparticle Zeta potential was measured (see below) using a Zetasizer Nano (Malvern Instruments Ltd., Worcester, UK).

### Nanoparticle release kinetics

Nanoparticle release kinetics and encapsulation efficiency were assayed in PBS, pH 7.4 as previously described^29,30^. Released NK2A peptide concentrations were quantified by micro bicinchoninic acid (microBCA) assay (Pierce Biotechnology, Rockford, IL). Kanamycin was quantified by *o*-pthalaldehyde derivatization^56^ (Fluoraldhehyde crystals, ThermoFisher Scientific, Carlsbad, CA) followed by absorbance spectroscopy at 340 nm (SpectraMax M3, Molecular Devices, San Jose, CA). Drug release kinetics are presented as fraction released, where the cumulative payload mass released is normalized by the total encapsulated payload mass.

### MDR *Salmonella* isolates and culture conditions

MDR *Salmonella* outbreak isolates (SX238, SX231, and SB395) as well as a non-MDR *Salmonella* isolate (SB377) used in this study are listed in Table 1. Bacterial freezer stocks were streaked individually onto Luria–Bertani (LB Lennox) agar (Sigma Aldrich) and incubated in a 37 °C incubator overnight. Bacterial cultures were prepared by growing each isolate from a single colony in 3 mL LB Lennox broth (ThermoFisher Scientific, Wilmington, DE) overnight at 37 °C in a shaker-incubator at 200 rpm.

### MDR *Salmonella*-NK-lysin peptide susceptibility assays

Overnight cultures of a MDR *Salmonella* outbreak isolate (SX238) and one non-MDR *Salmonella* isolate (SB377) were diluted 1:1000 in LB broth to obtain approximately 1 × 10^6^ colony forming units per milliliter (CFU/mL). One hundred microliters of each diluted culture were transferred into honeycomb® 100-well plates (~ 1 × 10^5^ CFU/well) (Growth Curves USA, Piscataway, NJ). Bovine NK-lysin peptides (NK1, NK2A, NK2B, and NK2C) were added at the final peptide concentrations of 20, 10, 5, 3.5, 2 µM in PBS (total volume = 20 µL) with a final bacterial-peptide volume of 120 µL/well. The plates were covered with lids and placed in an automated growth curve reader (Bioscreen C; Growth Curves USA, Piscataway, NJ) which was programmed for continuous shaking at 37 °C, and optical density readings at 600 nm (OD_600_) were recorded every hour for 24 h. Bacteria incubated with kanamycin and PBS were used as positive and negative control for the assay, respectively. The optical density data were analyzed, and growth curve graphs for each isolate were generated using MS Excel. Because NK2A peptide showed the strongest *Salmonella* growth inhibition, this peptide was selected for further studies. Twenty-four-hour growth curve assays for *Salmonella* isolate SX238 were repeated at different bacterial CFU (~ 1 × 10^5^, 1 × 10^6^, 1 × 10^7^, and 1 × 10^8^ CFU/mL) with NK2A (5 µM and 10 µM). Sc-NK2A was included as a control for the assay.

The bactericidal effect of NK2A peptide against MDR *Salmonella* (SX238) was also determined. Overnight SX238 culture was centrifuged (10,000 rpm for 2 min) and diluted 1:1000 in LB broth and/or PBS to obtain ~ 1 × 10^6^ CFU/mL; 100 µL of diluted bacterial culture was placed in a Honeycomb plate (~ 1 × 10^5^ CFU/well), NK2A (20, 5, 2 and 0 µM) was added and incubated at 37 °C in the reader with continuous shaking for 3 h. Bacteria in each well was 10 × serially diluted in LB broth, 10 µL was plated on Xylose-Lysine-Tergitol 4 (XLT4) (ThermoFisher) agar plates and incubated at 37 °C overnight. Bacterial colonies were enumerated for each NK2A dilution.

Antimicrobial activity of NK2A (final concentration ~ 5 µM) encapsulated nanoparticles (CPTEG:CPH, CPH:SA and CPTEG:SA) were tested against *Salmonella* isolate SX238 as previously described^32^. To prepare 10 µM NK2A stock solutions, required amounts of nanoparticles was measured, added into 1 mL PBS and briefly sonicated as previously described^32^. One hundred microliters of NK2A nanoparticles and 100 µL of diluted bacterial culture in PBS was added into a Honeycomb plate (~ 1 × 10^5^ CFU/well) and incubated at 37 °C in the reader with continuous shaking for 3 h. Bacteria in each well was 10 × serially diluted in LB broth, 10 µL was plated on XLT4 agar plates and incubated at 37 °C overnight. Kanamycin (final concentration = 16 µg/mL) encapsulated and empty nanoparticles were used as positive and negative control for the assay, respectively. Bacterial colonies were enumerated for each nanoparticle treatment.

The percentage of bacterial killing was calculated using the following Eq. (1 − [average number of live bacteria in NK2A-treated sample/average number of live bacteria in the negative control sample] × 100). The antimicrobial assays for *Salmonella* isolates were repeated at least three times in duplicate wells.

### Zeta potential measurements

*Salmonella* isolates were prepared for Zeta potential measurements via the diffusion barrier technique as previously described but with some minor modifications^35^. Briefly, overnight cultures of *Salmonella* in LB broth were centrifuged (10,000 rpm for 2 min), washed twice in Milli-Q water and resuspended in Milli-Q water (~ 1 × 10^9^ CFU/mL). Bacteria were then diluted 1:1000 in Milli-Q water supplemented with 10% PBS, then transferred to clear disposable folded capillary Zeta cells filled with 10% PBS via gel loading pipette tip (Malvern Instruments Ltd). The Zeta potential measurements (mV) of bacterial isolates were immediately measured using a Zeta potential analyzer equipped with He–Ne 633 nm laser (Zetasizer Nano, Malvern) at a constant voltage of 150 V at room temperature. The Zeta potential changes of *Salmonella* isolates following incubation with NK2A were also recorded. Bacterial isolates (~ 10^6^ CFU in 1 mL Milli-Q water) were incubated with 2 µM NK2A (in PBS) at 37 °C for 20 min. Capillary Zeta cells were washed with Milli-Q water between samples and refilled with Milli-Q water containing 10% PBS before measuring the Zeta potentials. Zeta potential changes of one *Salmonella* isolate (SB395) was also similarly tested with Sc-NK2A (2 µM) peptide. This method was also used for assessing NK-lysin peptide Zeta potentials. A total of five Zeta potential measurements of 40 runs each with 30 s cooling cycles for each sample was performed.

### Confocal laser scanning microscopy (CLSM)

The viabilities of bacteria following incubation with NK2A and Sc-NK2A were determined using culture-independent (function of the membrane integrity of the bacteria) staining technique by using LIVE/DEAD™ *Bac*Light™ bacterial viability kit (for microscopy) as described previously^20^. Bacteria in each treatment was visualized using a Nikon A1R + laser scanning confocal microscope (Nikon Instruments, Melville, NY) as described previously^20^. Images were obtained using plan Apo 60 × objective lens (oil) at numerical aperture 1.4. Final figures were prepared using Adobe Photoshop Elements 11.

### Transmission electron microscopy (TEM)

Morphological changes of bacteria following incubation with NK2A and Sc-NK2A peptides were studied by TEM. An overnight culture of *Salmonella* (isolate SX238) was centrifuged and resuspended in PBS. One hundred microliters of bacterial suspension were placed in a Honeycomb plate (~ 1 × 10^7^ CFU/well) and NK2A, Sc-NK2A (20 µM), and PBS were added and incubated at 37 °C with continuous shaking for 1 h. The bacterial suspensions were mixed with an equal volume of 3% glutaraldehyde in 0.1 M cacodylate buffer (pH 7.4) and processed for TEM as described before^24^. Sections were examined with a FEI Tecnai G2 Biotwin (FEI Co., Hillboro, OR) TEM and images were taken with Advanced Microscopy Technologies (AMT Inc., Danvers, MN) imaging camera.

### Propidium iodide (PI) uptake assay

The kinetics of NK2A-induced damage to *Salmonella* cell membranes were evaluated using PI uptake assay as described previously^24^. Briefly, an overnight culture of MDR *Salmonella* outbreak isolate SX238 was centrifuged, washed with PBS, and resuspended in PBS. Ninety microliters of bacterial suspension was transferred to a 96-well opaque plate (~ 1 × 10^7^ CFU/well), mixed with 10 µL of PI (final concentration = 3 µM) and incubated at 37 °C for 5 min. PBS, polymyxin B (2, 4, 25, 50 and 100 µg/mL), NK2A or Sc-NK2A (both at 2 and 5 µM) were added and PI fluorescent intensities in each well was continuously measured (15 s intervals, flex mode) using a FlexStation 3 multi-mode (fluorescent) microplate reader (Molecular Devices LLC., San Jose, CA) for up to 6 h. The excitation and emission wavelengths for PI was set up at 530 nm and 620 nm, respectively.

### NK2A-Lipopolysccharides (LPS) binding studies

The binding of NK2A to *Salmonella* LPS was studied by growth curve assay and TEM. Five micromolar NK2A (~ 2.2 µg/well) was preincubated with different amounts of *Salmonella enterica* serotype Typhimurium LPS (Sigma; LPS = 0, 0.5, 1.1, 2.2, 4.4, 8.8 µg/well, final volume of LPS and NK2A = 20 µL) at 37 °C in a honeycomb plate with continuous shaking for 30 min. Overnight *Salmonella* (isolate SX238) culture was diluted to ~ 1 × 10^6^ CFU/mL and 100 µL was transferred into wells containing NK2A-LPS and further incubated at 37 °C with continuous shaking for 24 h. The optical density (OD_600_) data were analyzed, and growth curve graphs were generated.

The direct binding of NK2A to *Salmonella* LPS/membranes was also examined by TEM using gold nanoparticle-conjugated NK2A (NK2A-AuNPs). Ten nanometer (diameter) NHS-activated gold nanoparticles (AuNPs) were conjugated to NK2A as described by the manufacturer (Cytodiagnostics Inc., Burlington, ON, Canada). One hundred microliters of *Salmonella* isolate SX238 was placed in a Honeycomb plate (~ 1 × 10^7^ CFU/well) and incubated with NK2A-AuNPs (20 µM) or AuNPs (without NK2A) at 37 °C with continuous shaking for 15–45 min. Bacterial samples were fixed with 3% glutaraldehyde and processed for TEM as described above.

### Statistical analyses

The data analysis for this paper was generated using SAS® software, Version 9.4 of the SAS System for Windows. Copyright © 2002–2012. SAS Institute Inc. SAS and all other SAS Institute Inc. product or service names are registered trademarks or trademarks of SAS Institute Inc., Cary, NC, USA. When significant, mean comparisons were performed using the predicted differences (PDIFF) option. Significant differences were established at *P* ≤ 0.05.

## Supplementary Information

Supplementary Information.

## Data Availability

All the data supporting the conclusions have been included within the article.
