# Supplementary material for: Bovine NK-lysin peptides exert potent antimicrobial activity against multidrug-resistant Salmonella outbreak isolates
Source: Sci Rep. 2021 Sep 29;11:19276. doi: 10.1038/s41598-021-98860-6 (PMC8481502; doi:10.1038/s41598-021-98860-6)
Supplement: Supplementary file 1 — Supplementary Information. [file 41598_2021_98860_MOESM1_ESM.pdf]

Supplementary information

Bovine NK-lysin peptides exert potent antimicrobial activity against multidrug-resistant *Salmonella* outbreak isolates

Rohana P. Dassanayake[1*], Briony M. Atkinson[2], Adam S. Mullis[3], Shollie M. Falkenberg[1], Eric M. Nicholson[4], Eduardo Casas[1], Balaji Narasimhan[3], Shawn M. D. Bearson[2*]

[1]USDA, Agricultural Research Service, National Animal Disease Center, Ruminant Diseases and Immunology Research Unit, Ames, IA, USA.
[2]USDA, Agricultural Research Service, National Animal Disease Center, Food Safety and Enteric Pathogens Research Unit, Ames, IA, USA.
[3]Department of Chemical and Biological Engineering and Nanovaccine Institute, Iowa State University, Ames, Iowa, USA.
[4]Virus and Prion Research Unit, National Animal Disease Center, Agricultural Research Service, United States Department of Agriculture, Ames, IA, USA.

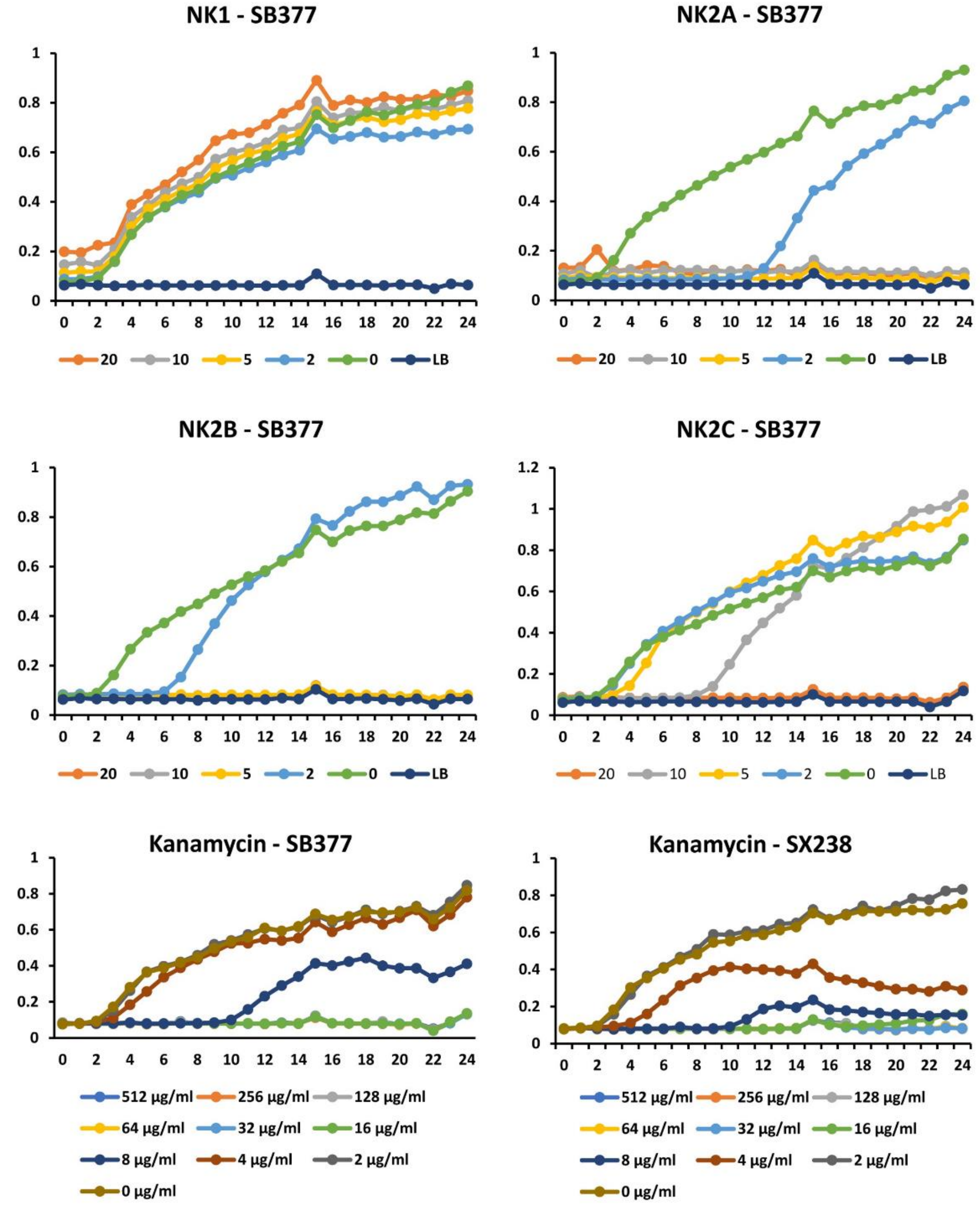

Supplementary Figure S1. Antimicrobial activity of bovine NK-lysin-derived peptides against a non-MDR *Salmonella*. A non-MDR *Salmonella* isolate SB377 was incubated with different concentrations of NK-lysin peptides (0, 2, 5, 10 and 20 µM; LB = Luria-Bertani (Lennox broth)), and optical density readings at 600 nm (OD_{600nm}) were recorded using an automated growth curve reader. Similarly, kanamycin sensitivity (0-512 µg/mL) of a MDR (SX238) and a non-MDR (SB377) *Salmonella* isolate was also studied. One representative growth curve from at least three independent experiments is shown.

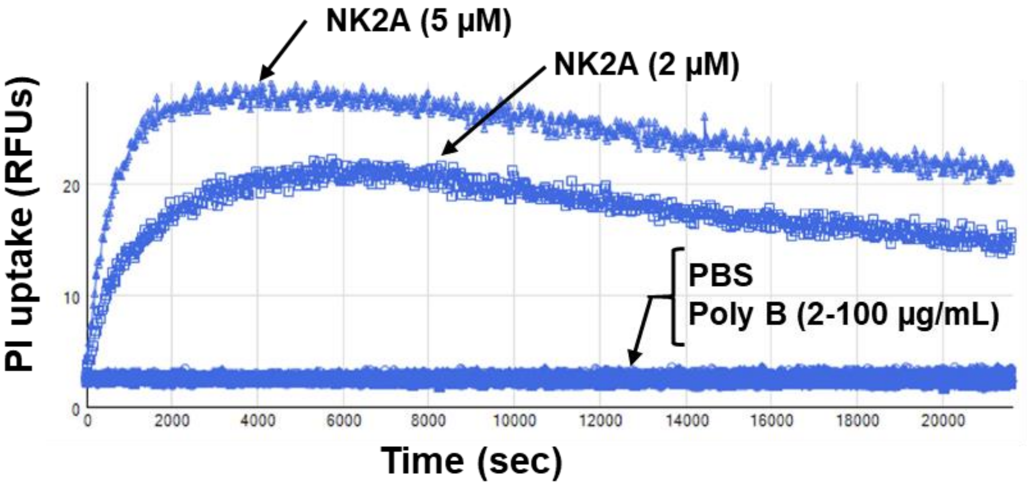

Supplementary Figure S2. Assessment of propidium iodide (PI) uptake by *Salmonella* incubated with NK2A and polymyxin B. *Salmonella* (SX238) was preincubated with PI followed by the addition of NK2A (2 and 5 µM final concentration) or polymyxin B (2, 4, 25, 50, and 100 µg/mL concentration), and PI signal was measured by a fluorescent microplate reader using flex mode with 15 sec intervals for up to 6 h. PI uptake signals are shown as relative fluorescent units (RFUs). Bacteria incubated with PBS was used as a negative control. One representative PI uptake graph out of three independent experiments is shown.

Authors' commentary: Lack of PI uptake signal in *Salmonella* following incubation with polymyxin B

Polymyxin B, a polycationic lipopeptide, is a bactericidal antibiotic that interacts with Gram-negative bacterial outer (LPS) and inner membranes[36]. Similar to NK2A, the cationic nature of polymyxin B facilitates the initial electrostatic attraction with anionic lipid A of LPS, thus inducing damage to the outer membrane while the disruption of inner membrane subsequently leads to bacterial death. Therefore, polymyxin B was selected as a positive control for the PI uptake assay. Although gradual increase of PI signal was observed with *Salmonella* (SX238) incubated with NK2A peptide within 1-2 minutes, such PI signal increase was not observed with polymyxin B or Sc-NK2A incubated samples. The lack of PI uptake signal increase with Sc-NK2A was not unexpected since Sc-NK2A showed minimal antibacterial activity (Fig. 2c and Fig. 3). However, the lack of PI uptake signal increase with polymyxin B was unexpected since polymyxin B was able to inhibit the growth of *Salmonella* (SX238) at $\geq$ 2 µg/mL concentrations in the growth curve assay. It is well known that the damage to bacterial membranes and bacterial death is directly proportionate to increasing polymyxin B concentration. Therefore, we tested a range of polymyxin B concentrations (2, 4, 25, 50 and 100 µg/mL) on SX238, but even the highest tested polymyxin B concentration (100 µg/mL) failed to induce PI signal increase during a 30 min incubation period. Although polymyxin B damaged the *Salmonella* outer membrane during the 30 min incubation, perhaps polymyxin B may need more time to induce damage to cytoplasmic membranes in order for PI to enter and bind with intracellular nucleic acids. To test this possibility, the PI uptake assay was continued for 6 h. Surprisingly, no PI uptake signal was observed with SX238 incubated with polymyxin B even up to 6 h with any of the concentrations tested. Therefore, the lack of PI uptake signal observed

with polymyxin B is not clear at this moment. Nonetheless, our PI uptake assay clearly demonstrated the rapid bacterial membranolytic nature of NK-lysins.
